# Proper Medical Education

**Published:** 1884-07

**Authors:** 


### PROPER MEDICAL EDUCATION.

In a paper read before the Medical Society of the State of Pennsylvania, May 14th, Henry Leffman, of Philadelphia, said:

"Higher specialization is the necessity of the time. The success which has been attained by dentistry shows that other departments, such as otology, laryngology, etc., might with advantage be pushed independently. There would be no reasonable objection to establishing the degrees of otology, ophthalmology, and so on, commensurate with the degree of Doctor of Dental Surgery."

